# PD-L1 expression on stromal tumor-infiltrating lymphocytes is a favorable prognostic factor in ovarian serous carcinoma

**DOI:** 10.1186/s13048-019-0526-0

**Published:** 2019-06-17

**Authors:** Ki Hyung Kim, Kyung Un Choi, Ahrong Kim, So Jung Lee, Jung Hee Lee, Dong Soo Suh, Byung-su Kwon, Chungsu Hwang

**Affiliations:** 1 Department of Obstetrics and Gynecology, Pusan National University Hospital, 179 Gudeok-ro, Seo-gu, Busan, 49241 South Korea; 2 Department of Pathology, Pusan National University Hospital, 179 Gudeok-ro, Seo-gu, Busan, 49241 South Korea; 3 Department of Pathology, Pusan National University Yangsan Hospital, 20, Geumo-ro, Mulguem-eup, Yangsan-si, Gyeongsangnam-do South Korea; 4 Research Institute for Convergence of Biomedical Science and Technology, 20, Geumo-ro, Mulguem-eup, Yangsan-si, Gyeongsangnam-do South Korea

**Keywords:** Ovarian cancer, Tumor-infiltrating lymphocyte, PD-L1, Survival

## Abstract

**Background:**

PD-L1 expression levels determined by immunostaining are known to be related to the survival rate and prognosis of patients with various types of cancers, as well as to the therapeutic response to immune checkpoint inhibitors. Recently, the U.S. Food and Drug Administration approved an immune checkpoint inhibitor for the treatment of non-small cell lung cancer along with the clones used for PD-L1 immunostaining to predict the resulting response. In this study, we performed PD-L1 immunostaining of tissue microarrays from ovarian epithelial cancer using SP263, an approved clone, and examined the effect of PD-L1 expression on survival rate and prognosis.

**Methods:**

Tissue microarrays were constructed from ovarian epithelial cancer tissues of 248 patients and PD-L1 immunostaining was performed using the SP263 clone. PD-L1 expression levels in tumor cells, intraepithelial tumor-infiltrating lymphocytes, and stromal tumor-infiltrating lymphocytes were evaluated, and the effect of PD-L1 expression on survival and prognosis was analyzed.

**Results:**

PD-L1 was detected in tumor cells as well as intraepithelial tumor-infiltrating lymphocytes and stromal tumor-infiltrating lymphocytes. It was most frequently expressed in stromal tumor-infiltrating lymphocytes. The Kaplan-Meier curve analysis and log rank test showed that only high stromal PD-L1 expression was associated with increased overall survival in ovarian serous carcinoma. Multivariate and univariate Cox regression analyses revealed that stromal PD-L1 expression was an independent prognostic factor, especially in ovarian serous carcinoma.

**Conclusions:**

PD-L1 immunostaining using SP263 was observed in tumor cells as well as intraepithelial and stromal tumor-infiltrating lymphocytes. PD-L1-expressing stromal tumor-infiltrating lymphocytes were associated with an increased overall survival rate and may serve as a favorable prognostic factor in ovarian cancer, particularly serous carcinoma.

**Electronic supplementary material:**

The online version of this article (10.1186/s13048-019-0526-0) contains supplementary material, which is available to authorized users.

## Introduction

Ovarian epithelial cancer is one of the most common cancers and a leading cause of death in women [1]. Despite standard treatment methods for chemotherapy following cytoreductive surgery, the mortality rate of ovarian epithelial cancer has not improved and new methods for predicting its prognosis and therapeutic strategies are required.

PD-1 is a transmembrane receptor of the Ig superfamily that lacks the relevant motif for binding to B7–1 and B7–2 and is expressed on thymocytes, mature T and B cells following activation, and on myeloid cells. PD-1 negatively regulates cytokine production and T cell proliferation by interacting with two ligands, PD-L1 and PD-L2. PD-1 and PD-1 ligands are involved in the induction and maintenance of peripheral tolerance [2]. Some studies have shown that tumor cells can also express PD-L1 and that tumor PD-L1 can interact with PD-1 on tumor specific T cells and suppress them to avoid host immune surveillance [3, 4]. Moreover, PD-L1 expression level was reported to be associated with patient survival rate and prognosis in various types of cancers, including ovarian cancer [5–10].

Recently, immune checkpoint inhibitors were approved by the U.S. Food and Drug Administration (FDA) for patients with advanced non-small cell lung cancer. Nivolumab and atezolizumab were approved for use in patients with advanced non-small cell lung cancer after failure of first-line therapy. Pembrolizumab was approved for use in patients with advanced non-small cell lung cancer for first-line treatment and for second-line treatment to include all patients with PD-L1-expressing non-small cell lung cancer. Clinical trials for these drugs showed that the level of PD-L1 immunostaining in tumor cells or tumor-infiltrating lymphocytes determined using specific clones such as 22C3, 28–8, SP263, and SP142, was correlated with drug efficacy and patient survival rate [11, 12]. However, there are no studies on the effect of PD-L1 expression determined using SP263, which is a PD-L1 clone predictive of immunotherapy response, on survival and prognosis in ovarian epithelial cancers. In addition, although some studies have identified the clinical significance of PD-L1 expression in ovarian cancer, it is unclear whether PD-L1 expression in any specific component of the tumor has a clinical significance.

In this study, we performed PD-L1 immunostaining of tissue microarrays from ovarian epithelial cancers using the SP263 PD-L1 antibody. We then evaluated PD-L1 expression in tumor cells, intraepithelial tumor-infiltrating lymphocytes, and stromal tumor-infiltrating lymphocytes separately and determined the effect of PD-L1 expression on the survival rate and prognosis of ovarian epithelial cancer.

## Materials and methods

### Patients

In total, 270 patients with primary epithelial ovarian cancer who underwent explorative laparotomy at the Department of Gynecology of Pusan National University Hospital from 1998 to 2013 were included in the study. All patients provided written informed consent and underwent surgery. We excluded patients who were not diagnosed with serous, mucinous, endometrioid, and clear cell carcinoma, and analyzed cases with available tissue slides from the cohort of all patients. After immunohistochemistry, we excluded cases in which both cores showed unreadable staining. As a result, 248 patients were included in this study. The biospecimens and data used for this study were provided by the Biobank of Pusan National University Hospital, a member of the Korea Biobank Network, which is supported by the Ministry of Health, Welfare and Family Affairs. All samples derived from the National Biobank of Korea were obtained with institutional review board approval.

All cases were examined by direct microscopic observation of hematoxylin and eosin staining of formalin-fixed and paraffin-embedded surgical specimens. Pathologic data including histological type, tumor grade, nuclear grade, mitosis, and tumor stage were obtained from the primary pathology reports. A retrospective review of electronic medical reports provided clinical information such as residual tumor and chemotherapy response. The histologic type and grade were determined according to the World Health Organization (WHO) classification, and tumor stage was determined according to the criteria of the International Federation of Gynecology and Obstetrics (FIGO). Tumor stage was reclassified as early in stage I and as advanced in stages II, III, and IV. Overall survival was measured from the date of surgery to the date of death or the last follow-up visit. The follow-up period ranged from zero to 206 months (median follow-up period, 61 months). The age of the patients varied from 15 to 70 years with an average age of 53.6 years. The clinicopathologic information including tumor grade, mitosis, nuclear grade, tumor stage, histologic type, residual tumor, and chemotherapy response is detailed in Table 1.

**Table 1:** The clinicopathologic characteristics of the patients

Clinicopathologic factors	Number of patients (%)
Histologic type	
Serous	140 (56.5)
Mucinous	47 (19.0)
Endometrioid	19 (7.7)
Clear	42 (16.9)
Residual tumor	
Optimal	167 (90.8)
Suboptimal	17 (9.2)
Tumor grade	
1	57 (23.0)
2	122 (49.2)
3	69 (27.8)
Tumor stage	
Early	68 (37.2)
Advanced	115 (62.8)
Nuclear grade	
Mild and moderate	137 (55.2)
Marked	111 (44.8)
Mitosis	
0–9/10 HPFs	93 (37.5)
10–24/10 HPFs	95 (38.3)
> =25/10 HPFs	60 (24.2)
Chemoresponse	
Regressive disease	73 (43.5)
Stable/progressive disease	95 (56.5)

### Tissue microarray and immunohistochemistry

Under a microscope, morphologically representative areas of tumors were selected and annotated on hematoxylin and eosin stained slides for each patient. Tissue cores of 2 mm in diameter were collected from the same areas of formalin-fixed and paraffin-embedded blocks, were annotated on the hematoxylin and eosin stained slides, and were rearranged in the recipient paraffin blocks. Tissue microarray blocks were sectioned at 4-μm thickness and immunohistochemical staining for PD-L1 on tissue microarray sections was performed using the BenchMark ULTRA automated staining platform (Ventana, Tucson, AZ) with anti-PD-L1 antibody (clone SP263, Ventana, Tucson, AZ). An OptiView DAB IHC Detection Kit (Ventana, Tucson, AZ) were used according to the manufacturer’s recommendations for the visualization of the primary anti-PD-L1 antibody and sections were counter-stained with hematoxylin.

### Immunohistochemical analysis

**Fig. 1:**
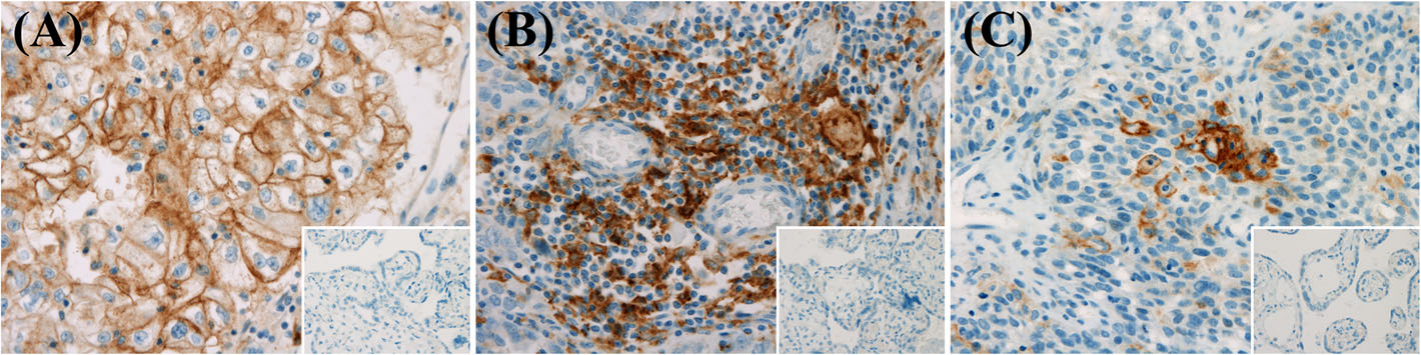
The representative cases of PD-L1 expression in tumor cell membrane (**a** magnification, × 400), stromal tumor-infiltrating lymphocytes (**b** magnification, × 400), and intraepithelial tumor-infiltrating lymphocytes (**c** magnification, × 400). Insets showed negative control for PD-L1 immunostaining (magnification, × 400)

The intensity and proportion of PD-L1 expression on tumor cells, stromal tumor-infiltrating lymphocytes, and intraepithelial tumor-infiltrating lymphocytes were analyzed separately. Both tumor cells and inflammatory cells were regarded as immunopositive if either the cytoplasm or membrane was stained. The intensities of PD-L1 staining in tumor cells, stromal tumor-infiltrating lymphocytes, and intraepithelial tumor-infiltrating lymphocytes were graded on a semiquantitative scale of 0 (none), 1+ (mild), 2+ (moderate), and 3+ (marked). The proportion of PD-L1 expression in tumor cells was the ratio of the area occupied by the tumor cells expressing PD-L1 in the tumor region composed of tumor cells. The ratio of the area occupied by immune cells showing PD-L1 positive in the tumor region composed of tumor cells was defined as the proportion of PD-L1 expression in intraepithelial tumor-infiltrating lymphocytes. The proportion of PD-L1 expression in stromal tumor-infiltrating lymphocytes was the ratio of the area occupied by the immune cells expressing PD-L1 in stroma. We considered high PD-L1 expression when the intensities of both tissue microarray cores were 2+ or greater, and the proportions of both tissue microarray cores were 5% of the area of tumor or stroma or greater. Representative cases of PD-L1 expression in the tumor, stromal tumor-infiltrating lymphocytes, and intraepithelial tumor-infiltrating lymphocytes are shown in Fig. 1.

### Statistical analysis

Chi-square test was used to identify the relationships between PD-L1 expression levels and clinicopathologic factors, and among stromal, tumor, and intraepithelial PD-L1 expression levels. Kaplan-Meier curve analysis and Log-rank test were performed to reveal the effect of PD-L1 expression on the overall survival rate. The prognostic effect of PD-L1 expression was evaluated using univariate and multivariate Cox regression analyses.

## Results

### Association between PD-L1 expression and clinicopathologic factors in all types of ovarian epithelial cancers and ovarian serous carcinoma

Spanning all histologic types of ovarian epithelial cancers, high PD-L1 expression in stromal tumor-infiltrating lymphocytes, tumor cells, and intraepithelial tumor-infiltrating lymphocytes was detected in 42 (16.9%), 21 (8.5%), and 26 (10.5%) patients of total 248 patients with ovarian epithelial cancers, respectively. Stromal PD-L1 expression levels were associated with histologic type (*p*-value = 0.015), residual tumor (*p*-value = 0.001), tumor grade (*p*-value < 0.001), and nuclear grade (p-value < 0.001). No relationship was identified between tumor PD-L1 expression and any of the clinicopathologic factors. Tumor grade (p-value < 0.001), nuclear grade (p-value = 0.004), and mitosis (p-value = 0.004) were associated with intraepithelial PD-L1 expression levels (Additional file 1: Table S1).

In ovarian serous carcinoma, high PD-L1 expression in stromal tumor-infiltrating lymphocytes, tumor cells, and intraepithelial tumor-infiltrating lymphocytes was noted in 29 (20.7%), 13 (9.3%), and 19 (13.6%) patients of total 140 patients with ovarian serous carcinoma, respectively. Stromal PD-L1 expression level was associated with tumor grade (*p*-value = 0.003) and nuclear grade (p-value < 0.001). Tumor PD-L1 expression was not associated with any clinicopathologic factors. Intraepithelial PD-L1 expression was associated with tumor grade (p-value < 0.001), nuclear grade (p-value = 0.004), and mitosis (p-value = 0.036) (Additional file 1: Table S2).

Stromal PD-L1 expression groups were positively correlated with intraepithelial PD-L1 expression groups. However, tumor PD-L1 expression groups were not associated with both stromal and intraepithelial PD-L1 expression groups (Table 2).

**Table 2:** The association among PD-L1 expression in each part of the tumor

		Intraepithelial PD-L1			Tumor PD-L1		
		Low expression (%)	High expression (%)	*p*-value	Low expression (%)	High expression (%)	*p*-value
Stromal PD-L1	Low expression	193 (93.7)	13 (6.3)	< 0.001	190 (92.2)	16 (7.8)	0.566
	High expression	29 (69)	13 (31)		37 (88.1)	5 (11.9)	
Intraepithelial PD-L1	Low expression				203 (91.4)	19 (8.6)	0.697
	High expression				24 (92.3)	2 (7.7)	

### Stromal PD-L1 expression is an independent prognostic factor in all histologic types of ovarian epithelial cancers and ovarian serous carcinoma

**Fig. 2:**
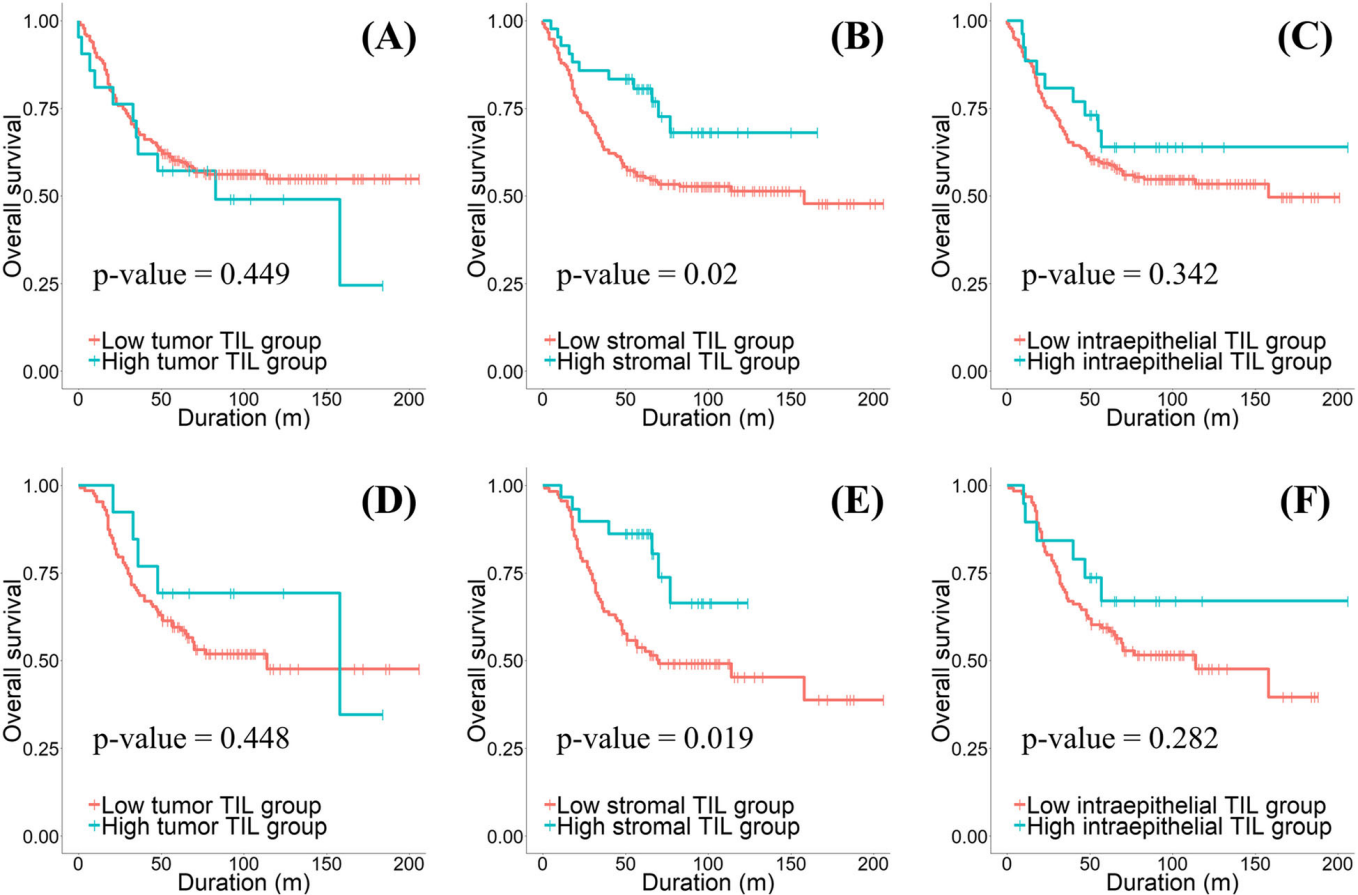
The results of Kaplan-Meier curve analysis and Log rank test for overall survival according to the tumor PD-L1 expression groups (**a**, *p*-value = 0.449), the stromal PD-L1 expression groups (**b** *p*-value = 0.020), and the intraepithelial PD-L1 expression groups (**c** *p*-value = 0.342) in all histologic types of epithelial ovarian cancers. The results of Kaplan-Meier curve analysis and Log rank test for overall survival according to the tumor PD-L1 expression groups (**d** *p*-value = 0.448), the stromal PD-L1 expression groups (**e** *p*-value = 0.019), and the intraepithelial PD-L1 expression groups (**f** p-value = 0.282) in ovarian serous carcinomas

Kaplan-Meier curve analysis and log rank test were performed to determine the effect of PD-L1 expression on survival. The results showed that only the high stromal PD-L1 expression group was associated with increased overall survival rate (*p*-value = 0.02) in all histologic types of ovarian epithelial cancers (Fig. 2a-c). In univariate and multivariate Cox regression analysis, stromal PD-L1 expression was an independent prognostic factor with residual tumor, tumor grade, and tumor stage (Table 3).

**Table 3:** Univariate and multivariate Cox regression analyses for overall survival in all types of EOCs

	Univariate		Multivariate	
	HR (95% CI for HR)	*p*-value	HR (95% CI for HR)	*p*-value
Stromal PD-L1				
Low expression	1 [reference]		1 [reference]	
High expression	0.484 (0.259–0.903)	0.023	0.291 (0.13–0.649)	0.003
Residual tumor				
Optimal	1 [reference]		1 [reference]	
Suboptimal	2.287 (1.206–4.337)	0.011	1.976 (1.016–3.845)	0.045
Tumor grade				
Grade 1	1 [reference]		1 [reference]	
Grade 2	3.83 (1.967–7.455)	< 0.001	2.026 (0.881–4.66)	0.096
Grade 3	3.428 (1.685–6.972)	0.001	2.469 (1.026–5.941)	0.044
Tumor stage				
Early stage	1 [reference]		1 [reference]	
Advanced stage	5.993 (3.079–11.67)	< 0.001	4.684 (2.318–9.466)	< 0.001

When the same analyses were performed on patients with ovarian serous carcinoma, only high expression of stromal PD-L1 was associated with increased overall survival rate and acted as an independent prognostic factor with residual tumor and tumor stage (Fig. 2d-f and Table 4).

**Table 4:** Univariate and multivariate Cox regression analyses for overall survival in ovarian serous carcinomas

	Univariate		Multivariate	
	HR (95% CI for HR)	*p*-value	HR (95% CI for HR)	*p*-value
Stromal PD-L1				
Low expression	1 [reference]		1 [reference]	
High expression	0.401 (0.183–0.883)	0.002	0.259 (0.09–0.739)	0.012
Residual tumor				
Optimal	1 [reference]		1 [reference]	
Suboptimal	2.389 (1.109–5.143)	0.026	2.294 (1.046–5.033)	0.038
Tumor grade				
Grade 1	1 [reference]		1 [reference]	
Grade 2	8.703 (1.198–63.22)	0.033	3.327 (0.448–24.73)	0.240
Grade 3	5.84 (0.783–43.55)	0.085	3.041 (0.396–23.35)	0.285
Tumor stage				
Early stage	1 [reference]		1 [reference]	
Advanced stage	15.39 (2.116–111.9)	0.007	12.27 (1.666–90.39)	0.014

## Discussion

Recent studies indicate that PD-L1 immunostaining is helpful in predicting patients with effective response to PD-1/PD-L1 immune checkpoint blockers [11, 13]. In addition to immune checkpoint inhibitors, PD-L1 immunohistochemistry assays corresponding to approved drugs such as the 22C3 clone for pembrolizumab, 28–8 for nivolumab, and SP142 for atezolizumab, were also approved. The SP263 immunohistochemical assay was also approved by the FDA as a complementary diagnostic tool for predicting patients with urothelial carcinoma who are expected to have a response to durvalumab. Although there are studies using several PD-L1 clones including SP142 [9, 10, 14, 15], this study is the first to demonstrate the clinical significance of PD-L1 immunostaining using the SP263 clone in ovarian cancer.

Hamanishi et al. [9] first performed PD-L1 immunostaining using their own clone and reported the clinical significance of PD-L1 expression in ovarian cancer. They only evaluated PD-L1 expression in tumor cells and classified the patients into high and low PD-L1 expression groups based on intensity alone. They showed that high PD-L1 expression was associated with increased overall and progression-free survival and PD-L1 was an independent, poor prognostic factor. Darb-Esfahani et al. [14] performed immunostaining for PD-L1 using the EPR1161 clone and interpreted PD-L1 expression in both tumor cells and tumor-infiltrating lymphocytes according to a semi-quantitative immune-reactivity score in high grade ovarian serous carcinoma. Immune-reactivity score is a scoring system that evaluates both intensity and proportion and they considered PD-L1 positive if the immune-reactivity score was 1 or greater than 1. The result showed that both the membranous tumor PD-L1 positive and tumor-infiltrating lymphocyte PD-L1 positive groups were associated with increased progression-free survival. However, only membranous tumor PD-L1 was an independent prognostic factor. Although they evaluated PD-L1 expression on tumor-infiltrating lymphocytes, it is uncertain whether they distinguished stromal and intraepithelial tumor-infiltrating lymphocyte. Webb et al. [10] performed PD-L1 immunostaining in ovarian cancer using two PD-L1 clones, SP142 and E1L3N. They classified patients with PD-L1 expression in more than one cell as a PD-L1 positive group and showed that PD-L1 positivity in high grade serous carcinoma was a favorable independent prognostic factor for disease-specific survival.

Although all of the above studies have shown that PD-L1 expression is associated with ovarian cancer prognosis, it is unclear whether PD-L1 expressed in any part of the tumor is associated with prognosis. We attempted to resolve this ambiguity by evaluating PD-L1 expression separately in tumor cells, intraepithelial tumor-infiltrating lymphocytes, and stromal tumor-infiltrating lymphocytes, and performed survival and Cox regression analyses for each. The results showed that only PD-L1 expression on stromal tumor-infiltrating lymphocytes was significantly associated with overall survival rate and acted as a favorable independent prognostic factor in all histologic types of ovarian cancers and ovarian serous carcinoma.

Theoretically, PD-L1 plays a role in inhibiting tumor-specific T cells, whether expressed in tumor cells or immune cells. Thus, the favorable prognostic effect of PD-L1-expressing stromal tumor-infiltrating lymphocytes is in contrast to the role of PD-L1. Webb et al. [10] revealed that the majority of PD-L1-positive tumor-infiltrating lymphocytes were tumor-associated macrophages. Liu et al. also showed that the majority of PD-L1-positive immune cells were CD68-positive macrophages and that they improved the overall survival rate and acted as a favorable prognostic factor in hepatocellular carcinoma [16]. We did not identify which cells in the stromal tumor-infiltrating lymphocytes were positive for PD-L1, but even though tumor-associated macrophages were the predominant PD-L1-positive cells in our results, it is difficult to explain the result because it has been known that PD-L1-expressing tumor-associated macrophages contribute to an immunosuppressive tumor microenvironment via inhibition of T cell proliferation and induction of T cell apoptosis to help tumor growth [17, 18]. Webb et al. explained that the favorable prognostic effect of PD-L1 expression in tumor-infiltrating lymphocytes is due to the adaptive resistance of activated T cells leading to a negative feedback mechanism in the tumor microenvironment [10]. Pulko et al. showed that upregulation of B7-H1 is essential for effector T cells to survive during the contraction phase of the immune response and to elicit protective immunity [19]. The study suggested that T cells expressing PD-L1 can directly overcome the immunosuppressive tumor microenvironment, explaining the favorable prognostic effect of stromal PD-L1 expression. Therefore, it is necessary to further study the more detailed components of stromal tumor-infiltrating lymphocytes and identify which subtype of stromal tumor-infiltrating lymphocytes expressing PD-L1 has clinical significance, especially a favorable prognostic effect in ovarian cancer.

Tang et al. revealed that PD-L1 expression on host cells, especially antigen-presenting cells, is essential for the response to PD-L1 blockade therapy in a murine model [20]. Heng Lin et al. also showed that PD-L1 expression on dendritic cells or macrophages was correlated with a response to the immunotherapy [21]. The results of these studies suggest that PD-L1 expression in tumor-associated macrophage is important for therapeutic effect of immune checkpoint inhibitors. As already mentioned above, Webb et al. and Liu et al. showed that the majority of PD-L1-positive immune cells were CD68-positive in ovarian cancer. The clinical trial of atezolizumab against various malignant tumors performed by Herbst et al. [11] showed that patients with high PD-L1 expression of tumor-infiltrating lymphocytes but not tumor as determined by immunostaining using the SP142 clone, were more responsive to atezolizumab compared to the patients with low PD-L1 expression of tumor-infiltrating lymphocyte. Therefore, PD-L1 expression level of tumor-infiltrating lymphocytes can be a predictive factor for the response to immunotherapy in ovarian serous carcinoma.

Our results showed that high stromal and intraepithelial PD-L1 expression were associated with increased tumor grade. These results suggest that high grade ovarian epithelial cancers are more easily detected in the host immune system, attracting more tumor-infiltrating lymphocytes and thus promoting PD-L1 expression in tumor-infiltrating lymphocytes due to adaptive resistance mechanism. PD-L1 expression in stromal tumor-infiltrating lymphocytes inversely correlated with tumor grade and had opposite effect on survival rate and prognosis of ovarian cancer patients. Nevertheless, in multivariate analysis of ovarian serous carcinoma, PD-L1 expression in stromal tumor-infiltrating lymphocytes was an independent prognostic factor, but not tumor grade was. This indicated that PD-L1 expression in stromal tumor-infiltrating lymphocytes may be very important factor in the prognosis of the patients with ovarian serous carcinoma.

Our results showed that the expression levels of stromal PD-L1 and intraepithelial PD-L1 were positively related to each other and were correlated with similar clinicopathologic factors. This may be due to the fact that accumulation of immune cells in the stroma is the pre-stage for immune cell invasion to tumor cell nests. Actually, high PD-L1 expression on intraepithelial tumor-infiltrating lymphocytes was also associated with increased overall survival with a looser cutoff value in this study and could be a prognostic factor for the response to immunotherapy. However, it was relatively easier to evaluate stromal tumor-infiltrating lymphocytes or PD-L1 expression than to evaluate intraepithelial tumor-infiltrating lymphocytes or PD-L1 expression in our experience. We thus suggest that the importance of stromal PD-L1 expression as a predictor for the response to immunotherapy needs to be explored further.

In conclusion, our study shows that PD-L1 expression on stromal tumor-infiltrating lymphocytes determined using the clone SP263 has a favorable prognostic effect and suggests that stromal PD-L1 could be a predictor for the response to immunotherapy in ovarian epithelial cancer, particularly in serous carcinoma.

## Additional file

Additional file 1: **Table S1.** The association between PD-L1 expression and clinicopathologic factors in all types of EOCs. **Table S2.** The association between PD-L1 expression and clinicopathologic factors in ovarian serous carcinomas. (PDF 334 kb)

## Data Availability

The datasets used and analyzed during the current study are available from the corresponding author on reasonable request.
