# Correction to: PD-L1 expression on stromal tumor-infiltrating lymphocytes is a favorable prognostic factor in ovarian serous carcinoma

**DOI:** 10.1186/s13048-019-0540-2

**Published:** 2019-07-23

**Authors:** Ki Hyung Kim, Kyung Un Choi, Ahrong Kim, So Jung Lee, Jung Hee Lee, Dong Soo Suh, Byung-Su Kwon, Chungsu Hwang

**Affiliations:** 1 Department of Obstetrics and Gynecology, Pusan National University School of Medicine, Biomedical Research Institute, Pusan National University Hospital, 179 Gudeok-ro, Seo-gu, Busan, 49241 South Korea; 2 Department of Pathology, Pusan National University School of Medicine; Biomedical Research Institute, Pusan National University Hospital, 179 Gudeok-ro, Seo-gu, Busan, 49241 South Korea; 3 Department of Pathology, Pusan National University Yangsan Hospital, 20, Geumo-ro, Mulguem-eup, Yangsan-si, Gyeongsangnam-do South Korea; 4 Research Institute for Convergence of Biomedical Science and Technology, 20, Geumo-ro, Mulguem-eup, Yangsan-si, Gyeongsangnam-do South Korea

**Correction to: J Ovarian Res (2019) 12:56**

**https://doi.org/10.1186/s13048-019-0526-0**

Following publication of the original article [1], the authors opted to correct the incorrect e-mail address of the corresponding author from 82-55-360-1850chungsu1982@gmail.com to chungsu1982@gmail.com, grant in the Acknowledgements section and Author details. Below are the updated version of the Acknowledgements and Author details.
