# Supplementary material for: PD-L1 expression on stromal tumor-infiltrating lymphocytes is a favorable prognostic factor in ovarian serous carcinoma
Source: J Ovarian Res. 2019 Jun 17;12:56. doi: 10.1186/s13048-019-0526-0 (PMC6580633; doi:10.1186/s13048-019-0526-0)
Supplement: Supplementary file 1 — Table S1. The association between PD-L1 expression and clinicopathologic factors in all types of EOCs. Table S2. The association between PD-L1 expression and clinicopathologic factors in ovarian serous carcinomas. (PDF 334 kb) [file 13048_2019_526_MOESM1_ESM.pdf]

Table S1. The association between PD-L1 expression and clinicopathologic factors in all types of EOCs

	Stromal PD-L1 expression			Tumor PD-L1 expression			Intraepithelial PD-L1 expression		
	Low expression (%)	High expression (%)	p-value	Low expression (%)	High expression (%)	p-value	Low expression (%)	High expression (%)	p-value
Histologic type			**0.015**			0.585			0.292
serous	111 (79.3)	29 (20.7)		127 (90.7)	13 (9.3)		121 (86.4)	19 (12.6)	
mucinous	45 (95.7)	2 (4.3)		43 (91.5)	4 (8.5)		44 (93.6)	3 (6.4)	
endometrioid	13 (68.4)	6 (31.6)		19 (100)	0 (0)		17 (89.5)	2 (10.5)	
clear	37 (88.1)	5 (11.9)		38 (90.5)	4 (9.5)		40 (95.2)	2 (4.8)	
Residual tumor			**0.001**			0.767			1.000
optimal	145 (86.8)	22 (13.2)		156 (93.4)	11 (6.6)		152 (91.0)	15 (9.0)	
suboptimal	9 (52.9)	8 (47.1)		15 (88.2)	2 (11.8)		15 (88.2)	2 (11.8)	
Tumor grade			**< 0.001**			0.277			**< 0.001**
1	52 (91.2)	5 (8.8)		55 (96.5)	2 (3.5)		54 (94.7)	3 (5.3)	
2	108 (88.5)	14 (11.5)		109 (89.3)	13 (10.7)		117 (95.9)	5 (4.1)	
3	46 (66.7)	23 (33.3)		63 (91.3)	6 (8.7)		51 (73.9)	18 (26.1)	
Tumor stage			0.496			0.428			1.000
early	59 (86.8)	9 (13.2)		65 (95.6)	3 (4.4)		62 (91.2)	6 (8.8)	
advanced	94 (81.7)	21 (18.3)		105 (91.3)	10 (8.7)		104 (90.4)	11 (9.6)	
Nuclear grade			**< 0.001**			1.000			**0.004**
mild and moderate	124 (90.5)	13 (9.5)		125 (91.2)	12 (8.8)		130 (94.9)	7 (5.1)	
marked	82 (73.9)	29 (26.1)		102 (91.9)	9 (8.1)		92 (82.9)	19 (17.1)	
Mitosis			0.068			0.891			**0.004**
0-9/10 HPFs	83 (89.2)	10 (10.8)		86 (92.5)	7 (7.5)		90 (96.8)	3 (3.2)	
10-24/10 HPFs	78 (82.1)	17 (17.9)		86 (90.5)	9 (9.5)		84 (88.4)	11 (11.6)	
>=25/10 HPFs	45 (75.0)	15 (25.0)		55 (91.7)	5 (8.3)		48 (80.0)	12 (20.0)	
Chemoresponse			0.164			0.167			0.279
regressive disease	57 (78.1)	16 (21.9)		65 (89.0)	8 (11.0)		64 (87.7)	9 (12.3)	
stable/progressive disease	83 (87.4)	12 (12.6)		91 (95.8)	4 (4.2)		89 (92.7)	7 (7.3)	

Table S2. The association between PD-L1 expression and clinicopathologic factors in ovarian serous carcinomas

	Stromal PD-L1 expression			Tumor PD-L1 expression			Intraepithelial PD-L1 expression		
	Low expression (%)	High expression (%)	p-value	Low expression (%)	High expression (%)	p-value	Low expression (%)	High expression (%)	p-value
Residual tumor			0.328			1.000			0.967
optimal	74 (83.1)	15 (16.9)		80 (89.9)	9 (10.1)		77 (86.5)	12 (13.5)	
suboptimal	8 (66.7)	4 (33.3)		11 (91.7)	1 (8.3)		11 (91.7)	1 (8.3)	
Tumor grade			**0.003**			0.449			< **0.001**
1	12 (100)	0 (0)		12 (100)	0 (0)		12 (100)	0 (0)	
2	66 (85.7)	11 (14.3)		70 (90.9)	7 (9.1)		73 (94.8)	4 (5.2)	
3	33 (64.7)	18 (35.3)		45 (88.2)	6 (11.8)		36 (70.6)	15 (29.4)	
Tumor stage			1.000			1.000			0.423
early	15 (78.9)	4 (21.1)		17 (89.5)	2 (10.5)		15 (78.9)	4 (21.1)	
advanced	67 (81.7)	15 (18.3)		74 (90.2)	8 (9.8)		73 (89.0)	9 (11.0)	
Nuclear grade			< **0.001**			1.000			**0.004**
mild and moderate	64 (92.8)	5 (7.2)		63 (91.3)	6 (8.7)		66 (95.7)	3 (4.3)	
marked	47 (66.2)	24 (33.8)		64 (90.1)	7 (9.9)		55 (77.5)	16 (22.5)	
Mitosis			0.204			0.412			**0.036**
0-9/10 HPFs	26 (83.9)	5 (16.1)		30 (96.8)	1 (3.2)		30 (96.8)	1 (3.2)	
10-24/10 HPFs	51 (83.6)	10 (16.4)		54 (88.5)	7 (11.5)		54 (88.5)	7 (11.5)	
>=25/10 HPFs	34 (70.8)	14 (29.2)		43 (89.6)	5 (10.4)		37 (77.1)	11 (22.9)	
Chemoresponse			0.128			0.759			0.102
regressive disease	31 (73.8)	11 (26.2)		37 (88.1)	5 (11.9)		34 (81.0)	8 (19.0)	
stable/progressive disease	45 (88.2)	6 (11.8)		47 (92.2)	4 (7.8)		48 (94.1)	3 (5.9)	
